# An Insight into Chemistry and Structure of Colloidal 2D-WS_2_ Nanoflakes: Combined XPS and XRD Study

**DOI:** 10.3390/nano11081969

**Published:** 2021-07-30

**Authors:** Riccardo Scarfiello, Elisabetta Mazzotta, Davide Altamura, Concetta Nobile, Rosanna Mastria, Simona Rella, Cinzia Giannini, Pantaleo Davide Cozzoli, Aurora Rizzo, Cosimino Malitesta

**Affiliations:** 1 Institute of Nanotechnology, Campus Ecotekne, CNR NANOTEC, via Monteroni, 73100 Lecce, Italy; riccardo.scarfiello@nanotec.cnr.it (R.S.); concetta.nobile@nanotec.cnr.it (C.N.); rosanna.mastria@nanotec.cnr.it (R.M.); aurora.rizzo@nanotec.cnr.it (A.R.); 2 Department of Biological and Environmental Sciences and Technologies, Campus Ecotekne, University of Salento, via Monteroni, 73100 Lecce, Italy; simona.rella@unisalento.it; 3 Institute of Crystallography, IC CNR, via Amendola 122/O, 70126 Bari, Italy; davide.altamura@ic.cnr.it (D.A.); cinzia.giannini@ic.cnr.it (C.G.); 4 Department of Mathematics and Physics “E. De Giorgi”, Campus Ecotekne, University of Salento, via Arnesano, 73100 Lecce, Italy; davide.cozzoli@unisalento.it; 5 UdR INSTM di Lecce, c/o, Campus Ecotekne, Universy of Salento, via Arnesano, 73100 Lecce, Italy

**Keywords:** transition-metal dichalcogenides, WS_2_ nanoflakes, WS_2_ colloidal nanocrystals, X-ray photoelectron spectroscopy, X-ray diffraction, Debye function

## Abstract

The surface and structural characterization techniques of three atom-thick bi-dimensional 2D-WS_2_ colloidal nanocrystals cross the limit of bulk investigation, offering the possibility of simultaneous phase identification, structural-to-morphological evaluation, and surface chemical description. In the present study, we report a rational understanding based on X-ray photoelectron spectroscopy (XPS) and structural inspection of two kinds of dimensionally controllable 2D-WS_2_ colloidal nanoflakes (NFLs) generated with a surfactant assisted non-hydrolytic route. The qualitative and quantitative determination of 1T’ and 2H phases based on W 4f XPS signal components, together with the presence of two kinds of sulfur ions, S_2_^2−^ and S^2−^, based on S 2p signal and related to the formation of WS_2_ and WO_x_S_y_ in a mixed oxygen-sulfur environment, are carefully reported and discussed for both nanocrystals breeds. The XPS results are used as an input for detailed X-ray Diffraction (XRD) analysis allowing for a clear discrimination of NFLs crystal habit, and an estimation of the exact number of atomic monolayers composing the 2D-WS_2_ nanocrystalline samples.

## 1. Introduction

Bidimensional transition-metal dichalcogenides (2D-TMDs) are nontoxic and earth abundant lamellar solids made of van-der-Waals (vdW) stacked atomic layers, with the general formula MX_2_ (e.g., M = Mo, W, Ti; X = S, Se, Te). Each individual layer of 2D-TMDs is three atom-thick, being the metal atom sandwiched between two chalcogens. Depending on the atomic stacking configuration, MX_2_ compounds can form different crystal structures. The most commonly encountered polymorphs are the hexagonal close packing and trigonal prismatic coordination (2H) phase and the tetragonal symmetry and octahedral coordination phase (1T) or its distorted polimorph (1T’) [1,2,3]. The properties of different polymorphs differ significantly from one another. For example, in the case of WS_2_, 2H-WS_2_ monolayers are semiconducting with a direct band gap of about 2 eV, whereas their 1T-WS_2_ and 1T’-WS_2_ counterparts possess a metallic character [4]. Moreover, in the form of ultrathin 2D nanostructures, in the mono- to few-layer regime, 2D-TMDs exhibit peculiar thickness-dependent properties, making them extremely attractive for several applications, spanning from optoelectronic and electronic devices to batteries and hydrogen-evolving photocatalysts [1,2,4,5,6,7,8,9,10,11].

Among various TMDs, WS_2_ attracts great attention for its plentiful prerogatives starting from large spin-orbit interaction [12] which offers a versatile platform for spin and valley physics studies as well as for being potentially employed for stimulated emission [13,14], microlasers production [15] and generally promising for ultrafast optics as a high-power flexible saturable absorber material. The application of a high-power pulsed laser [16] and innovative optoelectronic devices such as photodetectors [7] and light emitting transistors [17] may substantially benefit from 2D-WS_2_ features. Earth-abundant non-toxic 2D-WS_2_ with high concentration metallic edges has been reported as platinum-based co-catalysts efficient while cheaper competitor in hydrogen evolution electrocatalysis at low working over-potentials [18]. Besides electrocatalysis, 2D-WS_2_ nanosheets have been investigated for NIR light as well as strong UV-Vis light photocatalytic activity, indicating nanostructured 2D-WS_2_ as a valuable full solar-spectrum photocatalyst [19]. Moreover, solution dispersible 2D-WS_2_ based quantum dots, with an average size about 3 nm, due to their optical features, good cell permeability, and low cytotoxicity, have been indicated as promising and biocompatible probes for in vitro bio-imaging [20]. Thus, the versatility and the broad applicability have attracted the interest of the scientific community in 2D-WS_2_ nanostructures’ controllable production and characterization over the years.

Nowadays, one of the most intensively pursued goals towards device integration is attaining single to few-stacked atomic thick 2D-WS_2_ with solution processability, in order to obtain high quality thickness controllable sheets overtaking expensive vacuum depositions and substrate matched growth conditions which would eventually limit their mass fabrication [8]. The production of individually addressable, easily-processable 2D-WS_2_ with controllable and uniform sub-nanometer thickness and tailored lateral dimensions is very challenging. The currently available repertory of 2D-WS_2_ mostly comprises sheet-like nanostructures with irregular micrometer-scale lateral dimensions derived by liquid-phase exfoliation of corresponding bulk materials or by vapor-phase deposition on crystallographically oriented solid substrates [21].

Recently, wet-chemical synthetic approaches have emerged as powerful alternative routes to achieve morphologically and structurally controlled 2D-WS_2_ in the form of robust free-standing nanostructures with finely adjusted geometric parameters, stabilized by organic ligands bound to their surface/edges and, hence, sufficiently stable in the liquid phase to be safely manipulated and transferred to applications [22,23,24].

In particular, in ref. [22], two different amine-based non-hydrolytic chemical pathways have been reported for dimensionally controlled and solution processable 2D-WS_2_ nanocrystals generated in the mono-to few layers thickness regime. A step-by-step investigation of morphological, structural, optical, and chemical evolution of two breeds of 2D-WS_2_ nanoflakes generated into the two different amine-rich environments has been discussed. However, a more extensive and unambiguous chemical and structural understanding, based on X-ray Photoelectron Spectroscopy (XPS) and X-ray Diffraction (XRD), of as-prepared 2D-WS_2_ nanocrystals, is essential for an in-depth study of such materials and represents the object of the present work.

The XPS technique has been indeed successfully used for the study of the chemical composition of transition metal dichalcogenides for obtaining information about their synthetic processes and/or their proposed application. For example, the extent of sulfidation process of some transition metals after treatment with sulfur vapor has been quantified by XPS analysis [25]. It has been also used for demonstrating the effective electron transfer between the WS_2_ and the TiO_2_ through S-O-Ti bonding in 2D WS_2_ nanosheets decorated with TiO_2_ quantum dots used for ammonia gas sensing [26]. The content of amorphous WO_3_ in a few layer WS_2_ film has been evaluated by XPS analysis as well as its stabilization after the annealing procedure, allowing for postulating the role played by both WS_2_ and WO_3_ in the proposed gas sensing mechanism [27]. XPS analysis was also successfully used to compare the intrinsic air stability mechanism of several 2D transition metal dichalcogenides surfaces [28] evidencing different oxygen interaction processes at the basal plane and at the edges of the investigated materials, thus highlighting the importance of having controlled oxygen environment during crystal growth and defect passivation in order to provide high quality uniform materials for TMD-based device fabrication. In another work [29], the effect of the concentration of the reducing agent used during solution synthesis, 1,2-hexadecanediol, has been proven to significantly influence the final composition of TMDs. In some cases, XPS analysis has been also used to obtain structural information distinguishing 1T-WS_2_ and 2H-WS_2_ structure [30,31] on the basis of W 4f core level peaks.

The XPS characterization of 2D-WS_2_ NFLs that we report in the present work provides evidence on the W and S speciation giving additional information than expected based on the stoichiometric ratio. Also, information about the existence of two crystal phases, 1T/1T’ and 2H, has been inferred from XPS analysis. A single XPS peak for 1T/1T’ phase has been observed, in agreement with XPS data on other TMDs [32]. Moreover, the comparison of XPS results on two kinds of WS_2_ NFLs prepared under different alkylamines reaction environment is reported, revealing that the chemical nature of the alkylamines, although having a key effect in modulating the lateral size of the resulting nanoflakes, does not induce any variation of the atomic scale structure and chemical composition. It should be highlighted that the XPS technique can be considered in this case as a bulk technique due to the few monoatomic layer structure of 2D-WS_2_ NFLs thus enabling the investigation of the whole material thickness. Structural and chemical invariance are in turn confirmed through XRD characterization based on the Debye function calculation and are used as input data to reveal NFLs thickness variations. The full statistical representativity of sample features is achieved by the combination of the two non-local X-ray techniques, returning complementary volume averaged results.

## 2. Materials and Methods

### 2.1. Chemicals

Tungsten (VI) chloride (WCl_6_, 99.9%), 1-octadecene (C_18_H_36_ or ODE, 90%), oleyl amine (C_17_H_33_NH_2_ or OlAm, 70%), and octyl amine [CH_3_(CH_2_)_6_CH_2_NH_2_ or OctAm, 99%) were purchased from Sigma-Aldrich (Milan, Italy). All solvents used were anhydrous and of analytical grade. Chloroform, 2-propanol, and carbon disulfide (CS_2_) were purchased from Sigma-Aldrich (Italy). Dried acetone (max 0.0075% H_2_O) was purchased from SeccoSolv (Darmstadt, Germany). All chemicals and solvents were used as received without any further purification. OlAm and ODE were individually degassed at 80 °C for 1 h, then repeatedly purged with nitrogen and stored in a N_2_-protected glovebox prior to use.

### 2.2. WS_2_ NFLs Synthesis

2D-WS_2_ colloidal nanoflakes were synthesized as reported previously [22]. Briefly, 100 mg (0.25 mmol) of WCl_6_ were loaded in three-neck flask under N_2_ atmosphere in a glovebox and dispersed in 1 mL (3.1 mmol) of degassed ODE. Then, 1 mL (3 mmol) of degassed OlAm or a mixture of 1 mL (3 mmol) of degassed OlAm and 1 mL (6 mmol) of OctAm were used to growth, respectively, 2D-WS_2_ nanoflakes with lateral dimension <5 nm or ~25–30 nm. NFLs synthesized in only OlAm environment is denoted as sample A, while NFLs synthesized in OlAm/OctAm 1:2 molar ratio environment is denoted as sample B. OlAm and OlAm/OctAm mixture volumes were adopted to fully disperse WCl_6_ obtaining an initially dark suspension which consequently converted into an optically clear yellow/reddish solution by stirring the mixture at room temperature. The whole system was further degassed (20 mTorr) for 1h at 130 °C and, after backfilling the reaction environment with dried N_2_ gas, a solution of 60 μL (1 mmol) of CS_2_ in 2.5 mL (7.6 mmol) of degassed OlAm was swiftly injected into the mixture. The system was slowly heated (ramp rate of ~5 °C/min) to ~250 °C under N_2_ flow and aged at this temperature for 2 extra h. The reaction was quenched by removing the heating mantle and the solution was transferred at ~40–50 °C into N_2_-protected glovebox for nanocrystals purification. For the aid, 15 mL of dried 2-propanol/acetone mixture (2:1 *v*/*v*) were gently added to flocculate the 2D-WS_2_ NCs and discharge the unreacted reagents after centrifuging at 3000 rpm for 10 min. Two more cycles of dissolution in chloroform (or toluene) and re-precipitation with only acetone were also performed, obtaining fully dispersible samples in desired nonpolar solvents (toluene, chloroform), achieving an optically clear colloidal dark solution (sample A and sample B).

### 2.3. XPS Characterization

XPS measurements were recorded with an AXIS ULTRA DLD (Kratos Analytical, Manchester, UK) photoelectron spectrometer using a monochromatic AlKα source (1486.6 eV) operated at 150 W (10 kV, 15 mA). The base pressure in the analysis chamber was 5.3 × 10^−9^ torr. Survey scan spectra were recorded using a pass energy of 160 eV and a 1 eV step. High resolution spectra were acquired using a pass energy of 20 eV and a 0.1 eV step. In each case the area of analysis was about 700 μm × 300 μm. During the data acquisition a system of neutralization of the charge has been used. Processing of the spectra was accomplished by CasaXPS Release 2.3.16 software. The binding energy (BE) scale was referenced to the Au 4f_7/2_ peak at 84.0 eV. For the analysis of high resolution spectra all peaks were fitted using Shirley background and GL(30) lineshape (a combination of Gaussian 70% and Lorentzian 30%). For quantitative analysis, the relative sensitivity factors present in the library of CasaXPS for the areas of the signals were used. Surface charging was corrected considering adventitious C 1s (binding energies (BE) = 285 eV). For XPS analysis, samples were deposited by casting on an Au sheet.

### 2.4. XRD Characterization

XRD profiles were measured with a D8-Discover Bruker diffractometer (Bruker, Billerica, MA, USA) (2.2 kW) equipped with a CuKα (λ = 1.54 Ǻ) source (operated at 40/40 mA/kV), a Goebel mirror, a Eulerian cradle goniometer, and a scintillator detector. XRD patterns were collected at a fixed incident angle of *α*_i_ = 2° while moving the detector with a step size of 0.05° (2θ). Data were corrected for the fixed angular detector acceptance in the receiving slit detection mode, by multiplying the raw data by the width (*D*) of the projected illuminated sample area (*L*) at each 2θ angle: *D* = *L* × sin (π − *α*_i_ − 2θ), being all angles expressed in radians.

Samples were prepared by spreading concentrated chloroform solutions of the as-purified colloidal NFLs on a silicon substrate in a glovebox. The substrate was then transferred to the diffractometer. All samples were measured under an ambient atmosphere at room temperature.

### 2.5. TEM

TEM investigation was performed with a JEOL JEM 1400Plus microscope (Tokyo, Japan), equipped with a GATAN Orius SC600 CCD (Pleasanton, CA, USA) camera and a tungsten filament-source operating at 120 kV. Samples were prepared by dropping an as-synthesized WS_2_ nanoflakes dilute chloroform solution onto a carbon-coated copper grid.

### 2.6. UV-Vis Absorptions

UV-vis absorptions of freshly prepared and purified 2D-WS_2_ nanoflakes CHCl_3_ solutions were recorded using a Varian Cary300 spectrophotometer (Agilent Technologies, Torino, Italy).

## 3. Results

**Figure 1:**
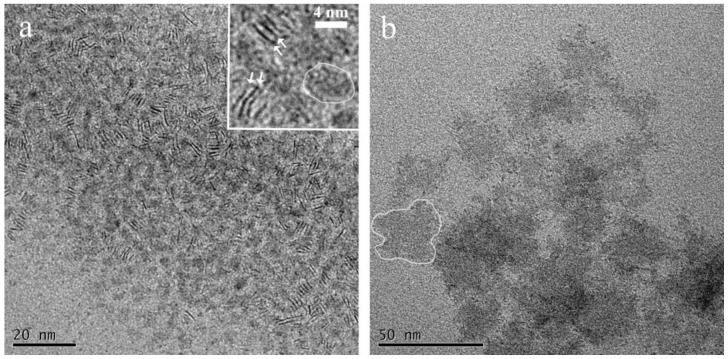
(**a**) Low resolution TEM of ~3–5 nm (sample A) generated in OlAm rich environment and (**b**) ~30 nm NFLs (sample B) generated in OlAm/OctAm 1:2 molar ratio environment.

We investigated solution casted 2D-WS_2_ colloidal nanocrystals that were directly generated in liquid media under surfactant assisted synthetic protocol. In particular, we compared two breeds of 2D-WS_2_ colloidal nanoflakes generated by a non-hydrolitic route in different high-boiling point alkylamine media reproduced from [22]. The different amine composition was found to be the key point to obtain a morphological and dimensional modification avoiding any other structural variation. Figure 1a,b reports low resolution TEM images of solution casted chloroform solution of ~3–5 nm (Figure 1a, sample A) and ~30 nm (Figure 1b, sample B) NFLs obtained in the different alkylamine environments. Both breeds of NFLs were characterized by an irregular and layered structure, hardly visible due to the low difference in contrast with carbon support grid in the low magnification TEM inspection (white line eye-guide in Figure 1a,b). Face-to-face stacked nanoparticles into short ribbon-like objects, which corresponded to the two-dimensional projections of nearly parallel standing nanocrystals, are evident in sample A (see white arrows in inset of Figure 1a).

### 3.1. XPS Characterization

**Figure 2:**
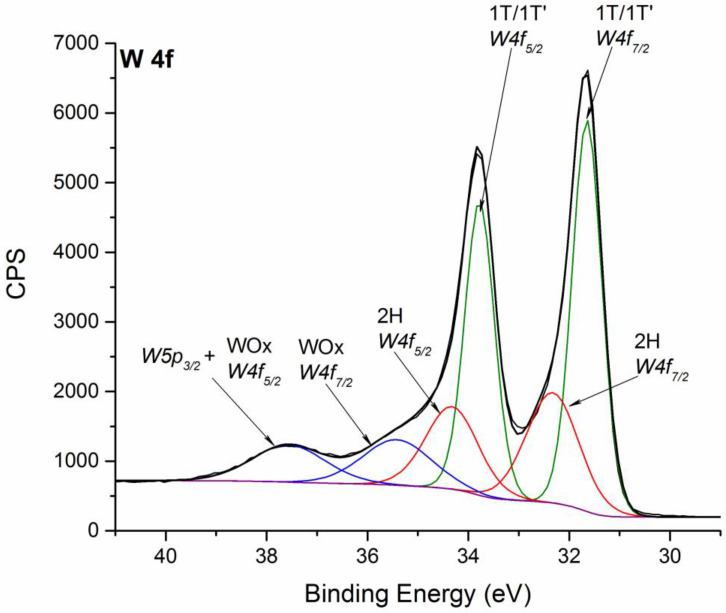
Fitted XPS spectrum of W 4f signal for WS_2_ NFLs sample B. Also, W 5p signal is reported. Spectrum is charging corrected. The same color is used for indicating doublet components.

The XPS detailed spectrum of W 4f signal of NFLs sample B is reported in Figure 2. As can be seen, the presence of both 2H and 1T/1T’ forms can be evidenced [6], with the latter originating a single peak, as previously reported for other TMDs [32]. 1T’-WS_2_ form is characterized by W 4f signal whose W 4f_7/2_ component is located at 31.6 eV. In the case of 2H-WS_2_ form, W 4f doublet is located at 32.3 eV (W 4f_7/2_). 1T’-WS_2_ represents evidently the most abundant form being its percentage equal to about (56.8 ± 5.4)%, while 2H-WS_2_ form represents about (27.0 ± 6.4)% of the investigated nanoflakes samples, as estimated by the area of XPS component signals. WS_2_ NFLs samples appear partially oxidized, as evidenced by the signals at 35.4 eV and 37.5 eV, attributable, respectively, to W 4f_7/2_ and W 4f_5/2_ of WO_x_ species. Note that the latter component is characterized by a higher area than what is expected for 4f_7/2_-4f_5/2_ doublet as it also includes the contribution from the W 5p_3/2_ component [18]. The presence of WO_x_ species could be due to post-synthesis surface oxidation [33] and represents (18.4 ± 2.1)% of the W 4f_7/2_ signal ascribed to 1T’ and 2H forms.

**Figure 3:**
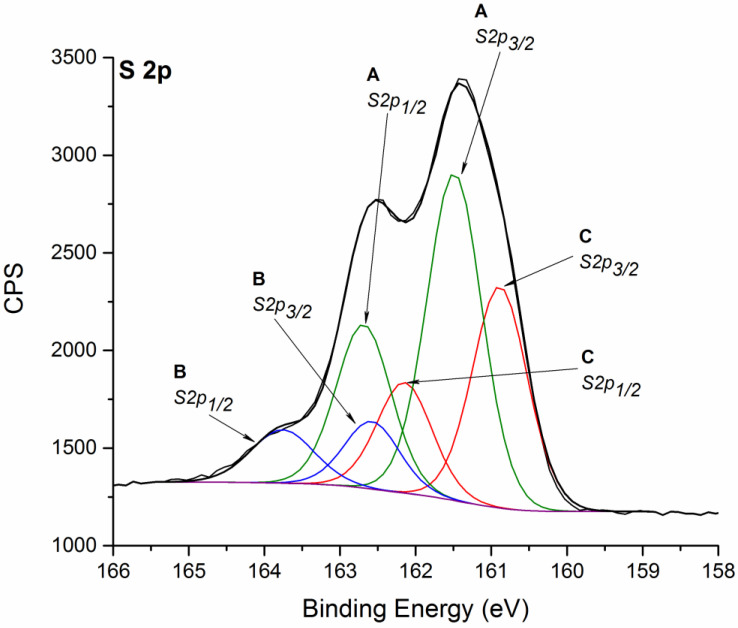
Fitted XPS spectrum of S 2p signal for WS_2_ NFLs sample B. Spectrum is charging corrected. Please see the text for the interpretation of peak doublet signals marked as A, B and C. The same color is used for indicating doublet components.

S 2p region (Figure 3) can be fitted into three components [31]. The intermediate doublet, denoted as “A” (S 2p_3/2_ at 161.5 eV), could be ascribed to sulfur S^2−^ ions in WS_2_. Species at higher and at lower binding energy (S 2p_3/2_ at 162.6 eV and 160.9 eV, denoted as “B” and “C”) could be instead ascribed to two kinds of sulfur ions, S_2_^2−^ and S^2−^, respectively, related to the formation of WO_x_S_y_ in a mixed oxygen-sulfur environment, likely associated with WO_x_ species identified in W 4f signal. It can be observed that the ratio between the doublet peaks of S 2p species at higher binding energy “B” is higher than the theoretical one (0.5). This could be due to the minor contribution of other sulfur species at higher oxidation states. The atomic ratio between S 2p and W 4f (considering the components related to 1T’ and 2H phases) is equal to 2.2, in good agreement with the theoretical one based on the stoichiometric ratio.

The reported interpretation of sulfur signal is in agreement with literature XPS data identifying a single sulfur species for both 1T/1T’ and 2H WS_2_ crystal phases [18,34]. In some other cases, two distinct S 2p components ascribed to 2H and 1T phases are individuated [35,36], but the quantitative estimation of their ratio is not in agreement with the ratio between 2H and 1T phases estimated on the basis of W 4f signal.

**Figure 4:**
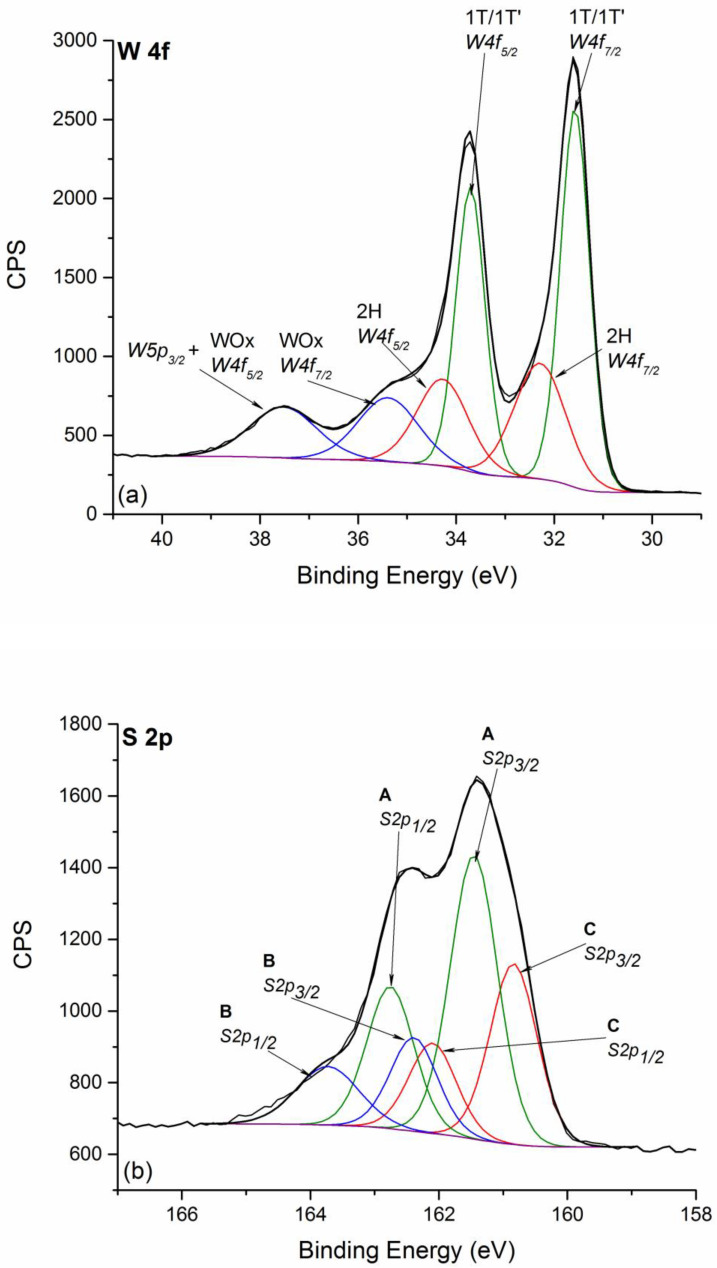
Fitted XPS spectra of W signal (**a**) and S 2p signal (**b**) for WS_2_ NFLs sample A. Spectra are charging corrected. In both spectra, the same color is used for indicating doublet components.

The XPS analysis of WS_2_ NFLs samples A shows the same components for W 4f (Figure 4a) and S 2p (Figure 4b) signals as for sample B. These results evidence the high similarity in chemical composition of the two synthesized samples thus showing that the chemical nature and steric hindrance of the alkylamines are essential to modulate the reactivity of such WS_2_ nanoclusters, which correlate with the lateral size of the resulting nanoflakes, but do not induce any variation in the atomic structure and chemical composition.

### 3.2. XRD Characterization

**Figure 5:**
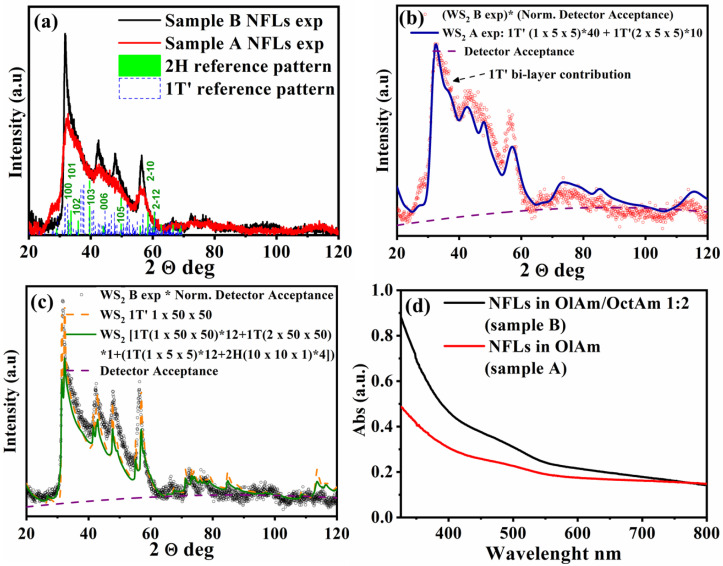
(**a**) Experimental XRD patterns (detector scans) of samples A (~3–5 nm NFls obtained in sole OlAm) and sample B (~30 nm NFLs obtained in OlAm/OctAm mixture in 1:2 molar ratio), with reference bars for 1T’ and 2H crystal phases at the bottom. Only 2H main reflections are indexed for clarity; (**b**) experimental XRD pattern for sample A, reproduced from panel (**a**), with relevant Debye function calculation; (**c**) experimental XRD pattern for sample B, reproduced from panel (**a**), with relevant Debye function calculation. (**d**) UV-Vis absorption spectra of ~3–5 nm (sample A, red) and ~30 nm NFLs (sample B, black) CHCl_3_ solutions.

Low magnification electron microscopy inspections reported in [22] evidenced that the different amine composition was found to be the key point to obtain a morphological and lateral dimensional modification avoiding any other evident structural variation. The obtained breeds NFLs presented in fact different lateral size (~3–5 nm for NFLs here denoted as sample A and ~30 nm for sample B) and were both characterized by irregular and ultrathin layered structure. Experimental XRD data (Figure 5a), analogous to those in Figures 1f and 4e of ref. [22], feature a convolution of multiple peaks, due to the characteristic broadening for nano-scale structures of the analyzed systems. The mixed nature of the samples, which can be referred to 1T’ and 2H phases, is visualized by the reference bars (dotted blue line for 1T’ and green solid line for 2H) at the bottom, and is demonstrated through the relevant Debye function calculations in Figure 5b,c, as it will be further discussed in detail. The coexistence of 1T’ and 2H phases with a given percentage mass ratio can be assumed based on XPS results, while the different features in the XRD patterns can be almost totally ascribed to NFLs thickness variations between samples A and B. Similarly, the undefined featureless optical profile of both samples, reported in Figure 5d, are also expected for a mixed semiconducting 2H and metallic 1T’ phase combination.

Additionally, XPS quantification allowed us to refine Debye function calculations introduced in a previous work [22] and aimed at a general characterization of the average structure of 2D NFLs, involving 1T’ and 2H crystal phases. Simulations of XRD patterns based on the Debye function allowed us to identify the main scattering contributions from NFLs with different size, shape and crystal structure (1T’ or 2H), identifying 1T’ as the main crystal phase in the samples, consisting in one or two monolayers thick NFLs, independently of other a priori information [22]. Being XRD a typically non-local characterization technique, these results were statistically representative of the whole sample volume. On the other hand, no differentiation could be assessed between samples A and B, due to the large number of variables taken into account. On the contrary, in the present analysis, we took advantage of XPS results by fixing the 1T’ to 2H mass ratio, while only allowing NFLs size variation. The correlation between XPS and XRD analyses is ensured by the non-local character of both techniques, providing averaged results from macroscopic sample volumes leading to high statistical significance and representativity. The comparison between calculated and experimental XRD patterns for samples A and B are reported and compared in Figure 5b,c, respectively. Such profiles were selected after calculating several scattering profiles as linear combinations of profiles relevant to NFLs with different size and structure, similarly to what reported in ref. [22] (Figures S1 and S5 in ref. [22]). It was found that essentially no scattering contribution from 2H structures was necessary to obtain a good agreement between experiment and calculations, which can be explained based on the reference bars reported at the bottom of Figure 5a. Such bars represent indeed the structure factors (relevant to 1T’ and 2H crystal structures) multiplied by the corresponding multiplicity factors, so as to obtain the final expected diffraction intensity: it can then be readily recognized that 1T’ structure features many more and closer diffraction peaks (blue bars) than 2H one (green indexed bars); furthermore, 2H XRD pattern features several high order diffraction peaks, the third index being the high order one, which are expected to be damped for a 2D lattice with its short dimension associated to the 00l reflections. As a consequence, the scattering contribution of 2H phase, also being a minority based on XPS results, is negligible compared to 1T’.

Thus, it has been found that all scattering features could be mainly described by the only 1T’ structure model in both samples: in particular, by 3 nm large and one or two monolayer thick NFLs in sample A (Figure 5b), and by 30 nm large and one monolayer thick NFLs in sample B (Figure 5c), expressed in terms of number of unit cells as 1 × 5 × 5 (monolayers, 80% relative fraction) or 2 × 5 × 5 (bilayers, 20% relative fraction) 1T’ unit cells in NFLs of sample A, and 1 × 50 × 50 (monolayers, 100%) 1T’ unit cells in NFLs of sample B. Minor discrepancies between experiments and calculations relevant to sample B can be ascribed to size dispersion and possibly to the weak 2H contribution.

## 4. Conclusions

A detailed investigation of the specific structural and chemical composition of 2D-WS_2_ colloidal nanoflakes (NFLs) was performed by a combination of X-ray Photoelectron Spectroscopy and X-ray Diffraction analysis based on the Debye function calculation. After previously demonstrating the ability of achieving NFLs with lateral size of ~3–5 nm and ~30 nm by using, respectively, a single oleyl amine or a mixture of oleyl amine and octyl amine, in the present work, the similarity in chemical composition and the thickness differences between the two synthesized samples was verified, simulated, and discussed. These results show that the chemical nature and steric hindrance of the alkylamines are essential to modulate the reactivity of forming WS_2_ nanoclusters, which correlate with the lateral size of the resulting nanoflakes and with a bilayer vs. monolayer thick 1T’ components in sample A (sample synthesized with only OlAm) and sample B (sample synthesized with OlAm:OctAm mixture) respectively. The effectiveness of XPS technique in providing information not only about chemical speciation but also about the existence of two crystal phases, 1T/1T’ and 2H, has been demonstrated, with the additional advantage of using XPS as a bulk technique in this case due to the few monoatomic layer structure of 2D-WS_2_ NFLs. By relying on XPS results, further structural details have been obtained through X-ray diffraction-based experiment and modeling, revealing precisely the number of monolayers in the 2D NFLs. High statistical significance and representativity of sample features is ensured by the non-local character of the XPS and XRD techniques.
